# Association between physical activity and mild cognitive impairment in community-dwelling older adults: Depression as a mediator

**DOI:** 10.3389/fnagi.2022.964886

**Published:** 2022-09-08

**Authors:** Xinya Liu, Yihua Jiang, Wenjia Peng, Meng Wang, Xiaoli Chen, Mengying Li, Ye Ruan, Shuangyuan Sun, Tingting Yang, Yinghua Yang, Fei Yan, Feng Wang, Ying Wang

**Affiliations:** ^1^School of Public Health, Fudan University, Shanghai, China; ^2^Key Laboratory of Health Technology Assessment, National Health and Family Planning Commission of the People’s Republic of China, Fudan University, Shanghai, China; ^3^Minhang District Mental Health Center of Shanghai, Fudan University, Shanghai, China; ^4^Fudan University Shanghai Cancer Center, Fudan University, Shanghai, China; ^5^Shanghai Municipal Center for Disease Control and Prevention, Shanghai, China; ^6^Shanghai Center for Clinical Laboratory, Shanghai, China

**Keywords:** mild cognitive impairment, physical activity, depression, mediating effect, influencing factors

## Abstract

**Introduction:**

Dementia has become a public health priority and is irreversible. Mild cognitive impairment (MCI), an intermediate state between normal cognition and dementia, is the prime time for early diagnosis and intervention. The activities of daily living of dementia patients are usually insufficient. Therefore, continuing to explore the risk factors of MCI, especially the influence of physical activity on MCI and its mechanism can enrich the relevant research fields in China.

**Materials and methods:**

For this cross-sectional study, 2,518 adults aged 60 years or older in Xinzhuang, Minhang District, Shanghai were recruited between July 2019 and April 2019, using a multistage, cluster-sampling method. A binary unconditional logistic regression model was used with MCI status as the dependent variable. Different types of physical activity were separately included in the multifactor model to test their correlations. Sensitivity analysis was performed using BADL as a stratification factor. The mediating effect of depression between physical activity and MCI was examined using the Bootstrap method.

**Results:**

This research includes 271 (10.8%) MCI. Old age (odds ratio 2.967 [95%CI 2.063∼4.269]), having diabetes (1.816 [1.302∼2.534]), and depression (3.012 [2.209∼4.108]) were possible risk factors for MCI. High education level (0.722 [0.523∼0.999]), medium level of physical activity (0.459 [0.326∼0.645]), and high level of physical activity (0.396 [0.270∼0.580]) were possible protective factors. Medium (0.548 [0.396∼0.757]) and high levels (0.557 [0.366∼0.846]) of physical exercise and medium (0.433 [0.318∼0.590]) and high levels (0.487 [0.296∼0.801]) of household chores are possible protective factors of MCI and their significance remained in the mutually adjusted model. Sensitivity analysis showed that physical activity and household chores were possible protective factors in all strata (*P* < 0.05). Physical exercise and work-related activities showed a protective effect in fully independent older adults, but the effect disappeared in those who cannot be fully independent. Depression played a partially mediating role with an indirect effect of 6.67%.

**Discussion:**

Overall, our results highlight that physical activity is a possible protective factor for MCI. Physical exercise and household chores have strong protective effects and future interventions could be targeted from this perspective. Depression plays a partially mediating role and more attention should be paid to the mental health of older adults.

## Introduction

Dementia is a progressive cognitive disorder characterized by a potential decline in cognitive function that commonly affects older adults and can have varying degrees of impact on the patient, to the point of disruption of daily life or even death (Writing Group of Expert Consensus on Long-term Healthcare of Cognitive Disorders in China, and Academy of Cognitive Disorder of China, 2016; Hill et al., 2017). Cognitive impairment can affect memory, learning, orientation, comprehension, judgment, calculation, language, visuospatial function, problem-analyzing, and solving abilities, and is often accompanied by psychiatric, behavioral, and personality abnormalities at some stage of the disease course (China Dementia and Cognitive Disorders Guidelines Writing Group, 2018a). Dementia can put a tremendous financial, mental, and physical strain on the patient and society.

The World Alzheimer Report 2015 shows that the prevalence of dementia was 46.8 million in 2015 and is expected to increase to 131.5 million by 2050, with most of the increase occurring in low and middle-income countries (Alzheimer’s Disease International et al., 2015). According to the latest estimates, China has the highest number of people with dementia in the world, with more than 15 million older adults having dementia, accounting for about 20% of the total number of people with dementia worldwide (Jia et al., 2020). The combined global cost of health care and lost revenue because of dementia has reached $81 billion per year and is expected to rise to $2.8 trillion by 2030 (Alzheimer’s Disease International, 2021). In a multicenter retrospective study for China, the annual socioeconomic burden of Alzheimer’s Disease (AD) for each patient amounted to $19,114.36 in 2015, with a total cost to society of $167.74 billion, equivalent to 1.47% of China’s domestic GDP, which is higher than the global average of 1.1% (Jia et al., 2018). It is foreseeable that, with the accelerated aging of the population, dementia will become a major public health problem in China in the future (Rizzi et al., 2014).

Dementia, as a central nervous system degenerative disease, is irreversible and currently has no effective cure, with a failure rate of 99.6% in clinical treatment trials over the past decade (Cummings et al., 2014). To prevent and intervene early in dementia, some scholars have proposed a precursor stage of dementia−mild cognitive impairment (MCI). MCI is defined as a progressive decline in memory or other cognitive functions but with essentially normal daily living skills and does not meet the diagnostic criteria for dementia (China Dementia and Cognitive Disorders Guidelines Writing Group, 2018b). MCI is likely to progress to dementia with attendant social and health impairment. Approximately 5−10% of MCI will progress to dementia each year and both MCI patients and those recovering from MCI have a higher risk of developing dementia than healthy older adults (Mitchell and Shiri-Feshki, 2009; Roberts et al., 2014). MCI is an intermediate state between normal cognition (NC) and dementia and neurological degeneration can still be reversed through neuroplasticity. Thus, interventions for MCI patients are of greater public health significance than for dementia patients (Huang et al., 2005).

Physical activity is an effective way for older adults to maintain their physical and mental health and is also inextricably linked to their cognitive function (Fan, 2019). The protective effect of moderate to vigorous physical activity on cognitive decline is significant (Guure et al., 2017). However, although different types of physical activity have different effects on cognitive function, there is a general paucity of related studies. Physical exercise and household chores have a protective effect on cognitive decline, but work-related activity has no significant effect on cognitive function (Dupré et al., 2019).

Mental health has a strong correlation with cognitive dysfunction such as MCI, and older adults with depression are at a higher risk of developing MCI (Li et al., 2018). Depression also has more influencing factors, such as gender, age, and education level. Among these factors, studies have confirmed that physical activity has a preventive effect on depression (Petersen, 2016). Both physical activity and depression have effects on cognitive function, and physical activity interacts with depression (Lindwall et al., 2011), this indicates that depression may play a mediating role in the effect of physical activity on cognitive function, which has been verified in a few studies (Spirduso, 2009; Vance et al., 2016).

However, the mediating mechanism of depression between physical activity and MCI is less explored in China and needs to be confirmed by more evidence. The purpose of this study is to understand the current situation of MCI among the community-dwelling older adults in Xinzhuang Town, Minhang District, Shanghai, China, as well as the physical activity and other possible risk factors for MCI. Additionally, it explores the mediating effect of depression between MCI and physical activity. Based on this, we aim to provide scientific and credible evidence for the prevention and early intervention of MCI to maintain older adults’ physical and mental health and improve their quality of life.

## Materials and methods

### Study design

This is a cross-sectional, home-based questionnaire-type study. It began in July 2019 and ended in August 2019. This study investigates several factors associated with MCI including socio-demographic variables, diseases, behavioral variables, and depression. The sociodemographic variables include gender, age, education, and residence; the diseases include hypertension, diabetes, and other diseases associated with MCI; and the behavioral variables include smoking, alcohol consumption, and physical activity. The dependent variables are cognitive status and daily living ability.

### Study participants

The study population was older adults aged 60 years and above who usually lived in Xinzhuang Town, Minhang District, Shanghai, and was selected by a multi-stage cluster random sampling. When calculating the sample size, combining the results of international studies, the specificity with significance test level at 0.05 is generally 70% and above. The estimated prevalence of MCI in Chinese older adults aged 60 years and above is 15.5%, so the allowable error is 0.015 (Jia et al., 2020). Further, considering a 10% missing rate, the expected sample size was determined to be 2,500 after rounding.

The neighborhoods in Xinzhuang town were selected randomly and the older adults were stratified by age. One of the 53 neighborhoods in Xinzhuang was first selected, if the population does not meet the needs of the sample, the next neighborhood is randomly selected until the number meets the requirement. Finally, 2,518 older adults participated in this study.

The inclusion criteria for the participants were: (1) age ≥ 60 years; (2) resident older adults in the Xinzhuang Town area of Minhang District (≥6 months of residence); (3) informed and consenting to the research; and (4) conscious, articulate, and with sufficient ability to cooperate with the research. The exclusion criteria were: (1) older adults who did not live in the community or were separated from their registered permanent residence during the research period; (2) people who were blind, aphasic, or severely hearing impaired; (3) people with severe diseases that could affect brain function or cognitive function; (4) people with a severe perceptual impairment that prevented them from completing cognitive function measurements; (5) people who were at the end-stage of life or had other conditions that prevented them from completing the questionnaire.

This research protocol was approved by the Ethical Review Board of Fudan University (reference number: IRB#TYSQ 2019-2-03), and informed written consent was obtained before data collection.

### Measures

In this study, the AD-8 scale, which is a subjective assessment tool, was used to assess the cognitive status of older adults in the sample community, while the BADL scale was combined to determine the basic life skills of older adults. An older adult was considered to have MCI if he or she was conscious with no obvious signs of dementia, if the AD-8 test showed abnormalities, and if the BADL score was greater than 60, which means they are capable of basic self-care in daily living.

The AD-8 can be administered either as an informed person-based scale or as a self-assessment scale. The AD-8 was effective as a self-assessment scale when applied to Chinese older adults, with a sensitivity of 85.7%, and specificity of 77.6%, and has great potential for application in primary health services (Mao, 2014). In this study, Cronbach’s alpha coefficient was 0.679 (greater than 0.6) with acceptable reliability.

The BADL scale, also known as the Barthel Index, published by Barthel in 1965, is a scale used to measure activities of daily living with good reliability and validity and is widely used to measure older adults (Zhou et al., 2019). Since the capacity for daily activities significantly affects the physical activity of older adults, the BADL is used as a stratification factor in this study and older adults with a score of 100 will be considered fully independent, while those with scores less than 100 will be classified as not fully independent. In this study, Cronbach’s α coefficient is 0.901, which is greater than 0.8 indicating very good reliability.

Physical activity was measured using the Physical Activity Scale for the Elderly (PASE), which is designed specifically for older adults and has been widely validated and used in China and abroad. A study found that the validity of the PASE scale was 0.51 and the reliability was 0.90 when it was used to measure the physical activity of Chinese older adults, which is considered high reliability (Yu and Qiu, 2014). The PASE includes 3 sub-dimensions as physical exercise, household chores, and work-related activities, and the scoring is based on a weighted sum of 11 activities. The scale score generally ranges from 0 to 400, and sometimes exceeds 400, with a higher score associated with a higher level of physical activity. In this study, the scores of the 3 sub-dimensions were calculated separately and summed to calculate the total score.

This study used the Geriatric Depression Scale (GDS-30) to assess depression in older adults. The scale score ranges from 0 to 30, with a higher score associated with more severe depressive symptoms. The multiple types of reliability of the GDS-30 were found to perform well in most studies and the scale has been widely used in older adults with accurate results (Guo et al., 2019). In the exploratory analysis of this study, the older adults with scores of 0–10 were classified as non-depressed and those with scores of 11−30 were classified as depressed. In the mediated effects analysis, the scores were defined as continuous variables. In this study, the Cronbach’s α coefficient of the scale was 0.860, which is greater than 0.8 indicating high reliability.

### Statistical analysis

Continuous variables were presented as mean values (± SD) and compared using the Student’s *t*-test between groups. The Pearson χ^2^-test was used to check the independence between categorical variables.

The association between physical activity and MCI was assessed using several binary logistic regression models. The full model logistic regression was used to examine the effect of each factor on MCI (for all ordered categorical variables, the lowest levels were adopted as reference categories). Further, the statistically significant variables in the univariate analysis were used as adjustment variables, and two binary logistic regression models were used to analyze the association of MCI with physical activity and its different types. Based on the full model logistic regression, sensitivity analysis was performed using BADL as a stratification variable. *P*-values less than 0.05 were considered statistically significant.

The mediating role of geriatric depression in physical activity and MCI was examined using the Bootstrap method. All analyses were conducted in SPSS version 26.0, MPLUS version 8.0, and plotted with R version 4.1.0.

## Results

### Participant characteristics

Characteristics of the participants are shown in Table 1. The study groups did not differ regarding gender, pension, or the percentage of alcohol consumption (all *P* > 0.05). More people in the MCI group were older than 80 years old (43.2%) than in the NC group (12.7%). The proportion of older adults with lower education levels was higher in the MCI group (73.1%) than in the NC group (60.3%). The MCI group has more older adults living alone (11.8%) than the NC (7.1%) group. More older adults in the MCI group had hypertension, diabetes, and depression (all *P* < 0.001) but fewer smoked (*P* < 0.05). The older adults in the MCI group performed worse than the NC group in both the total physical activity score and each dimension score (all *P* < 0.001).

**TABLE 1:** Characteristics of the sample.

Variables	Features	NC, *n* (%) (*n* = 2,247)	MCI, *n* (%) (*n* = 271)	*P*-value
Gender	Male	1,000(44.5)	113 (41.7)	0.380
	Female	1,247(55.5)	158 (58.3)	
Age	60∼70	1,224(54.5)	81 (29.9)	**<0.001**
	70∼80	737 (32.8)	73 (26.9)	
	≥80	286 (12.7)	117 (43.2)	
Education	Junior high school and below	1,355(60.3)	198 (73.1)	**<0.001**
	High school and above	892 (39.7)	73 (26.9)	
Residence	Living with others	2,088(92.9)	239 (88.2)	**0.005**
	Living alone	159 (7.1)	32 (11.8)	
Pension	<2,000	282 (12.6)	45 (16.6)	0.11
	2,000∼5,000	1,564(69.6)	186 (68.6)	
	≥5,000	401 (17.8)	40 (14.8)	
Hypertension	No	1,327(59.1)	118 (43.5)	**<0.001**
	Yes	920 (40.9)	153 (56.5)	
Diabetes	No	1,954(87.0)	204 (75.3)	**<0.001**
	Yes	293 (13.0)	67 (24.7)	
Smoking	No	1,893(84.2)	243 (89.7)	**0.019**
	Yes	354 (15.8)	28 (10.3)	
Alcohol consumption	No	1,879(83.6)	237 (87.5)	0.104
	Yes	368 (16.4)	34 (12.5)	
Depression	No	2,006(89.3)	175 (64.6)	**<0.001**
	Yes	241 (10.7)	96 (35.4)	
Physical activity	Low level	709 (31.6)	172 (63.5)	**<0.001**
	Medium level	751 (33.4)	57 (21.0)	
	High level	787 (35.0)	42 (15.5)	
Physical exercise	Low level	904 (40.2)	177 (65.3)	**<0.001**
	Medium level	838 (37.3)	62 (22.9)	
	High level	505 (22.5)	32 (11.8)	
Household chores	Low level	755 (33.6)	173 (63.8)	**<0.001**
	Medium level	1,130(50.3)	77 (28.4)	
	High level	362 (16.1)	21 (7.7)	
Work-related activity	No	2,066(91.9)	266 (98.2)	**<0.001**
	Yes	181 (8.1)	5 (1.8)	

NC, normal cognition; MCI, mild cognitive impairment. Bolded P values represent results that are statistically significant.

### Association between physical activity and mild cognitive impairment

**FIGURE 1:**
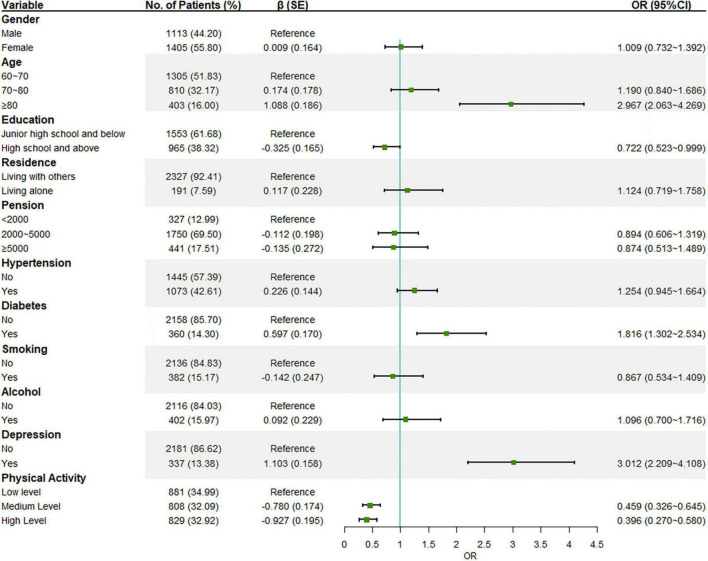
Logistic regression with MCI screening results as the dependent variable.

With MCI screening results as the dependent variable, all collected variables were included-gender, age, education, residence, pension, hypertension, diabetes, smoking, alcohol consumption, and physical activity (PASE total score) (Figure 1). Individuals over 80 years old (*P* < 0.001) and who had diabetes (*P* < 0.001) or depression (*P* < 0.001) had an increased risk of developing MCI. On the contrary, the analysis highlighted that a high educational level (*P* = 0.049) and medium (*P* < 0.001) or high level of physical activity (*P* < 0.001) were associated with no MCI, and the higher the level the more pronounced the protective effect.

**FIGURE 2:**
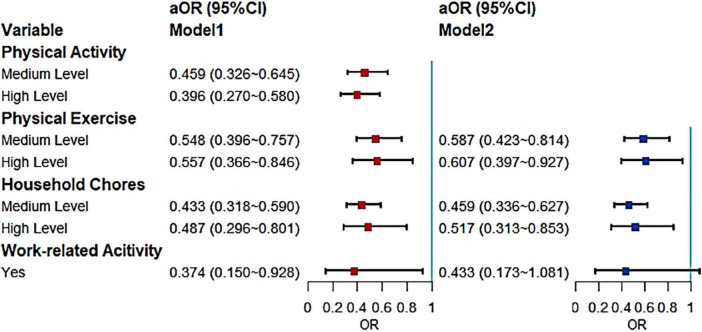
Effects of different types of physical activity on MCI (aOR, adjusted odds ratio; Use the low level and negative response as the reference).

The statistically significant variables in the univariate analysis were used as adjustment variables (including gender and age as *a priori* variables) and a binary logistic regression model was used to examine the association between MCI screening results and physical activity and different types of physical activity (Figure 2). The results obtained after four regressions of different dimensions of physical activity were run separately as Model 1. Based on Model 1, different dimensions of physical activity are co-incorporated into Model 2 for mutual adjustment.

Model 1 shows that physical activity in older adults was a protective factor for MCI; those with higher levels of physical activity were less likely to develop MCI. Further, the differences between medium (*P* < 0.001) and high level physical activity (*P* < 0.001) were statistically significant. In each dimension, both medium (*P* < 0.001) and high levels (*P* < 0.001) of physical exercise had a significant protective effect on MCI in older adults. Medium (*P* < 0.001) and high levels (*P* = 0.005) of household chores were also protective factors, but the protective effect of higher levels in both physical activity and household chores was less pronounced than the medium levels. The adjusted results for physical exercise and household chores remained the same in Model 2. For work-related activity, although also shown to be a protective factor in Model 1 (*P* = 0.034), the significance disappeared in the reciprocal adjustment of Model 2.

**FIGURE 3:**
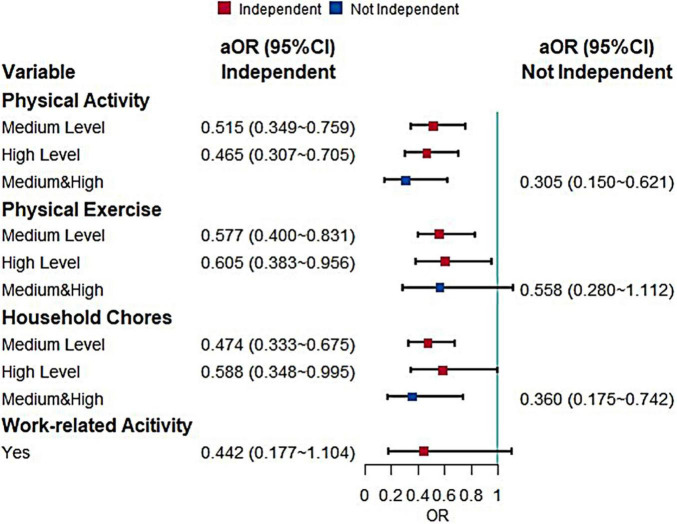
Effects of different types of physical activity on MCI when stratified by BADL (aOR, adjusted odds ratio; Use the low level and negative response as the reference).

The next logistic regression used BADL as a stratifying factor to analyze the effect of physical activity on MCI (Figure 3). There were 2,219 independent older adults and 299 older adults who are not fully independent. Regarding the total level of physical activity, there was a protective effect for fully independent older adults and similar results were observed for the other sub-dimensions. Both medium (*P* = 0.003) and high levels (*P* = 0.031) of physical exercise and medium (*P* < 0.001) and high levels (*P* = 0.048) of household chores were protective factors for MCI in fully independent older adults. There was no effect of work-related activity on the older adults developing MCI, but the *P*-value was close to 0.06 (*P* = 0.08).

Due to the small number of older adults who were not independent, there were too few observations of physical activity and its sub-dimensions, so the medium and high levels were combined in the statistical analysis, while the work-related activity was not included in the multifactorial analysis because it appeared to have zero observations. It was found that medium and high levels of physical activity (*P* = 0.001) and medium and high levels of household chores (*P* = 0.006) can reduce the risk of developing MCI in older adults who are not fully independent.

### Mediating effect of depression

The bootstrap method was used to test the mediating effects, with a sampling value set at 2,000, physical activity as the independent variable, geriatric depression as the mediating variable, and MCI screening results as the outcome variable. Additionally, gender was included in the model as an *a priori* variable, as well as age and education, which have a clear effect on MCI, as adjustment variables. The results are shown in Table 2. The direct and indirect effects of depression in the relationship between physical activity and MCI were significant (the 95% CI for the odds ratio does not contain 1), with odds ratios of 0.933 and 0.514 for the indirect and direct effects, respectively. This indicates that depression mediated the pathway and played a partially mediating role with its indirect effect accounting for −0.003/−0.045 × 100% = 6.67% of the total effect.

**TABLE 2:** Mediating effect of depression in the physical activity-MCI.

	Estimate (95%CI)	OR (95%CI)
Indirect effect	−0.003 (−0.005, −0.002)	0.933 (0.915, 0.949)
Direct effect	−0.042 (−0.051, −0.033)	0.514 (0.444, 0.599)
Total effect	−0.045 (−0.054, −0.036)	0.479 (0.415, 0.554)
Prop. mediated	6.67%	

Gender, age, and education as adjusted variables.

## Discussion

This cross-sectional study included 2,518 community-dwelling older adults to clarify the prevalence of MCI in the community, re-explore the factors influencing MCI, discover the relationship between different types of physical activity and MCI, as well as verify the partially mediating role of depression in the relationship between physical activity and MCI.

The AD-8 scale combined with the BADL scale was used to detect the prevalence of MCI in older adults; the results showed that the prevalence of MCI in older adults in Xinzhuang Township was 10.8%. The prevalence of MCI in most international studies is concentrated between 15 and 18% in developed countries such as the United States (Petersen et al., 2014), the United Kingdom (Richardson et al., 2019), and Germany (Luck et al., 2007), and the latest authoritative study in China reported a similar prevalence (15.5%) (Jia et al., 2020). Although the prevalence detected in this study was low compared to this interval, it was generally consistent with the interval of 6.1–30.4% in low and middle-income countries (McGrattan et al., 2021). The large difference in prevalence between this study and others may be due to variations in research methods, instruments, and criteria (Guo et al., 2019), or it may be that the older adults have difficulty in detecting symptoms of their cognitive decline, and therefore the detection rate is underestimated (Huang et al., 2005). Although this research showed a slightly lower prevalence of MCI among older adults in the Shanghai community than in other studies, the nearly 11% prevalence still needs to be taken seriously by the relevant authorities. According to the Shanghai Bureau of Statistics, the proportion of the household population aged 60 years and above in Shanghai has exceeded 35% as of 2019 (Shanghai Municipal Health Commission, 2020). The national level of aging will increase in the future, combined with the conversion rate of 53% of MCI progressing to dementia over 3 years, if effective early interventions are not taken, the future burden of dementia will be unimaginable (Hanninen et al., 2000). The current situation urgently requires the early identification of risk factors for MCI and effective interventions for those older adults who still have a chance of MCI reversal to cope with the serious challenges of upcoming aging.

This study found that age, education, diabetes, depression, and physical activity influenced MCI. Age is by far the greatest identified risk factor for cognitive impairment such as MCI (Dunne et al., 2021) and the prevalence of MCI increases significantly with advancing age. As the brain functions gradually decline with age, the accumulation of negative changes in brain structure and function predisposes older adults to an accelerated decline in cognitive function (Mozley et al., 1999; Fratiglioni et al., 2010). The effect of education on MCI was also observed in this study where older adults with higher levels of education were less likely to develop MCI, which aligns with previous studies (Sattler et al., 2012; Ding et al., 2015). This may be attributed to education’s ability to effectively increase the cognitive reserve in the brain, thus, allowing for more leeway in cognitive decline during old age (Mungas et al., 2018). Data from the *China Quality of Life Development Report for the Elderly (2019)* revealed that the number of older adults with elementary school education and below is extremely high in China, exceeding 70%. Therefore, it is important to design appropriate interventions for older adults with low education levels. This study found that people with diabetes had a higher risk of developing MCI, supporting the results obtained in other studies (Luchsinger et al., 2007; Cheng et al., 2012; Xiu et al., 2019). There is a strong link between blood supply and brain function (Galiano et al., 2020), and negative changes or shocks from this link are a key cause of MCI (Nelson et al., 2016). Here, diabetic hyperglycemia may negatively affect this link, accelerating cognitive decline in the brain (Yu et al., 2019). Numerous studies have shown that depression is a major risk factor for MCI in older adults (Geda et al., 2006; Li et al., 2018), and similar results were obtained in this study. Data from a prospective cohort in China showed that middle-aged and older adults who are depressed at baseline have a more rapid decline in cognitive status and that timely and effective prevention and treatment can reduce the decline in cognitive function (Yang et al., 2020). Depression and cognitive dysfunction often coexist in the same patient (Mukku et al., 2021), and one study found that geriatric depression and Alzheimer’s disease share three neurobiological features including neurodegeneration, cerebrovascular dysfunction, and elevated levels of neuroinflammation (Weisenbach et al., 2019).

Several previous studies have shown that physical activity reduces the risk of cognitive disorders such as MCI in older adults (Geda et al., 2010; Wang et al., 2013), and this study’s results are similar. Additionally, this study found that different types of physical activity also have a protective effect on MCI. Among them, physical exercise and household chores remained significant in protecting against MCI after adjusting for possible confounders and mutual adjustment of different types of physical activities. However, physical exercise became insignificant in a multifactorial analysis among older adults who were not fully independent, while the significance of work-related activity disappeared in the mutually adjusted multifactorial analysis and this result aligns with other studies (Dupré et al., 2019). The failure of work-related physical activity to provide comparable protection may be because although work is also a physical activity, it is seen as compulsory and motivated by others, and work does not distract people from stress or improve their sense of self-esteem but is more likely to bring stress (Asztalos et al., 2009). The impact of physical activity in older adults who are not fully independent was not significant in the multifactorial analysis. This may be because there were fewer individuals under stratification, resulting in insufficient statistical validity; thus, studies should be conducted for such older adults in the future. This study suggests a protective effect of household chores on cognitive function, which has been confirmed by many studies (Dupré et al., 2020b), including one showing that household/transportation-type physical activity significantly reduces the risk of dementia in older adults (Dupré et al., 2020a).

This study validated a potential partial mediating role of depression between physical activity and MCI, with the effect being approximately 6.67%, which is consistent with existing studies’ results (Vance et al., 2005; Spirduso, 2009). One study observed a physical activity-depression-cognitive function pathway with higher levels of physical activity being associated with fewer depressive symptoms, which, in turn, were associated with better cognitive performance (Vance et al., 2016). The observed mediating effect of depression was 23.8% in a Chinese study and is higher than in this study (Chen et al., 2018), possibly due to differences in the measurement instruments used. Further, the AD-8 scale used in this study was unable to provide information on the cognitive functioning subdomain; so, some information may have been missing. It has also been proposed that physical activity positively affects emotional, mental, and motivational states, while depression, for example, negatively affects such states, so the offsetting effect of the two reflects physical activity affecting cognitive function through depression (Spirduso, 2009). Many scholars also explore the influence of other psychosocial-behavioral mechanisms on the relationship between physical activity and cognitive function (Stillman et al., 2016), such as sleep and social support (Bherer et al., 2013; White et al., 2017), which should be the focus of future research.

This study has some limitations. First, it is a cross-sectional study and its inherent limitations make it necessary to be cautious about the causal and mediating effects of the study. A longitudinal cohort study may be considered in the future to clarify the causal association between physical activity and depression. Second, because the study used the AD-8 questionnaire to evaluate cognition in older adults, it was not possible to examine the influence factors on specific functional domains of cognition and this should be considered in the future. Third, this study took the form of self-report of physical activity, which may introduce varying degrees of recall bias. More accurate measurements of physical activity should be made in the future using objective measures. Finally, brain activity was not explored in this study and the effect of this aspect should be further considered.

## Conclusion

This study found that people who are older, less educated, living alone, and suffering from diabetes are more likely to develop MCI. The results suggest that the focus should be on this vulnerable group. Further, they should be treated with specific attention to their physical and mental status, especially their cognitive status, and given more care and support outside of the conventional prevention and treatment measures. This study’s results showed that not only does physical exercise have a protective effect on the cognitive function of older adults but household chores also have a similar protective effect. Therefore, it is recommended to encourage different types of physical activities in older adults through various means. The present study also observed a mediating relationship between depression in physical activity and MCI, suggesting that older adults with depressive symptoms can reduce their risk of developing MCI through active physical activity interventions.

## Data availability statement

The datasets presented in this article are not readily available because Data sharing is not applicable to this article due to privacy restrictions. Requests to access the datasets should be directed to corresponding author.

## Ethics statement

The studies involving human participants were reviewed and approved by the Ethical Review Board of Fudan University (reference number: IRB#TYSQ 2019-2-03). The patients/participants provided their written informed consent to participate in this study.

## Author contributions

XL and YJ contributed to methods, data curation, and writing – original draft preparation. FW and YW contributed to the research design, conceptualization, and writing – review and editing. TY, YY, and FY partially participated in the research design. WP, MW, XC, and ML participated in the data curation and investigation. All authors have read and approved the final version of the manuscript, and agreed with the order of authorship.
